# Participatory eco-landscape design: the case of NRIAG eco-park in Helwan, Egypt

**DOI:** 10.1186/s44147-021-00012-0

**Published:** 2021-10-06

**Authors:** Alia Sameh Okasha, Asmaa Aly El Mekkawy

**Affiliations:** 1 A visiting lecturer at the College of Engineering and Technology, Department of Architectural Engineering and Environmental Design, the Arab Academy for Science, Technology & Maritime Transport (AASTMT), Heliopolis, Cairo, Egypt; 2 Urbanist and Independent researcher, Cairo, Egypt

**Keywords:** Multidisciplinary, Eco-landscape, Multi-methods, Participatory, Digital tools, Design process, Helwan, Egypt

## Abstract

As cities get more crowded and polluted, eco-landscape design gains increasing attention. Open spaces play a vital role in healing the natural environment as well as the physical and mental health of the citizens. This paper presents an exploratory eco-park design project in Helwan, Egypt. The project focuses on the opportunity of integrating marginalised natural environments, such as Wadis (dry streams), with the urban fabric through Eco-landscape design. The current work explores the complex environment, characterised by detailed multidisciplinary data, which requires multi-layer analysis. The discussion evaluates the tremendous effect of integrating the participatory qualitative method with other analytical and digital tools, such as modelling and Geographic Information Systems (GIS), to deduce scientific details and activities in the preliminary phases of zoning plans. This results in a constructive framework for merging these multi-methods and tools within the participatory eco-landscape design process. In addition, the conclusion highlights the peculiarity of the eco-landscape design and practice in the current Egyptian situation in a broad sense.

## Introduction

Climate change, unhealthy living in dense urban environments, food insecurity, water scarcity, biodiversity loss, ecosystems’ degradation and people’s disconnection from nature are challenging issues that are being faced worldwide. There has been noticeably more press concerning health issues and public open spaces to respond to post-pandemic emergent needs, where international competitions and increased calls for research about public space projects are detected [1, 2].

The field of eco-landscape has overgrown during the past two decades. It is an intersection of many disciplines that involve diverse components, such as economics and sociology, the earth sciences and geography, remote sensing and computer applications [3]. Such a comprehensive nature of eco-landscape has much to offer for solving the emergent socio-environmental problems. Today, designers and planners recognise the necessity of developing an adequate understanding of ecology to intervene innovatively and intelligently on existing natural systems [4, 5].

Muller [6] argues that this shift starts by understanding that ecosystems can flourish in cities, if the way they are built is retrofitted. Thus, instead of doing a “less harmful” effect on the environment (similar to the sustainability approach), we should “maximize the good” [6]. This approach does not favour specific design aspects over another; rather, it tends to reveal as many layers of information as possible to develop the most efficient solutions.

Due to this new approach, many landscape designers are shifting their practice from picturesque and highly maintained landscape areas towards the use of living and eco-machines [7], constructed/designed ecologies [5], green-blue infrastructures [8, 9], landscapes of survival [10], and nature-based solutions [11] to provide more resilient natural areas inside the cities. As declared by Stokman [12], this perception would shift the profession from designing for privileged communities to solving urbanisation-related problems.

The emergent eco-landscapes depend on natural processes to deliver various ecosystem services like carbon sequestration, noise and urban heat island reduction, flood mitigation, and wastewater treatment, besides delivering other psychological, spiritual, and health benefits. Accordingly, eco-landscapes hold great potential in moderating urban poverty’s environmental and social consequences in marginalised communities [13]. They are very beneficial in areas disconnected from central systems, or where the urban grid confronts noticeable topographical change, such as Wadis. However, producing properly designed interventions to benefit both urbanity and nature is a challenge due to inadequate knowledge of ecosystems’ complexity. This lack of knowledge could be reduced by synthesising the “small-space ecology” scattered between fields [14]. This includes the site’s physical characteristics like microclimate, hydrology, and soil type, along with ecologic characteristics like species distribution and plant types, where science-based solutions are produced instead of relying on designers’ preferences.

While eco-landscape design practice evolves internationally, developing countries still struggle to research and practice proper eco-landscape design [15]. In Egypt, the pressing issue of water scarcity has gained increased attention because of the escalated water losses of the Nile surface evaporation, ineffective traditional irrigation methods, the increased water demand, and the construction of the Grand Ethiopian Renaissance Dam. Furthermore, climate change, urban compactness, lack of green open spaces, financial limitations, and many other challenges raise the demand for more ecological methods to approach landscape design. Therefore, investigating and integrating new methods is necessary to be aligned with this environmental, economic, and social complexity, particularly when the diverse background, local knowledge, and landscape design practice fail to give a comprehensive understanding of local ecologies and their potential role as city infrastructures.

The paper sets the criteria of the case study selection, exploring its potentials and the scope of this research. Then, the tactics and methods of the research and design process are elaborated, where integrating digital tools with participatory approaches helps deduce more scientific details. The research concludes with a framework for integrating these tools within the eco-landscape design process in the current complex situation in developing countries.

### Case study selection criteria

As noticed, Wadis (dry urban streams) present rare ecological opportunities in the poor dense fabric of Cairo. Dry urban streams are hardly being recognised by local urbanists as natural open spaces in the city because the streams’ studies are scattered between different disciplines like hydrology, geology and ecology. This research presents Wadis’ potential and defines possible guidelines, methods and framework that may shape the future eco-landscape practice in a specific selected site. Nevertheless, this framework can be generalised and applied to other similar sites. The selection criteria address the following:

- The study area is located inside an urban fabric interrupted by a dry Wadi.
- The project is within a polluted urban context, where introducing new green open spaces is needed.
- Finding a site unconnected to the central sewage system will allow more integration of urban and natural systems.

### Helwan district in Egypt

**Fig. 1:**
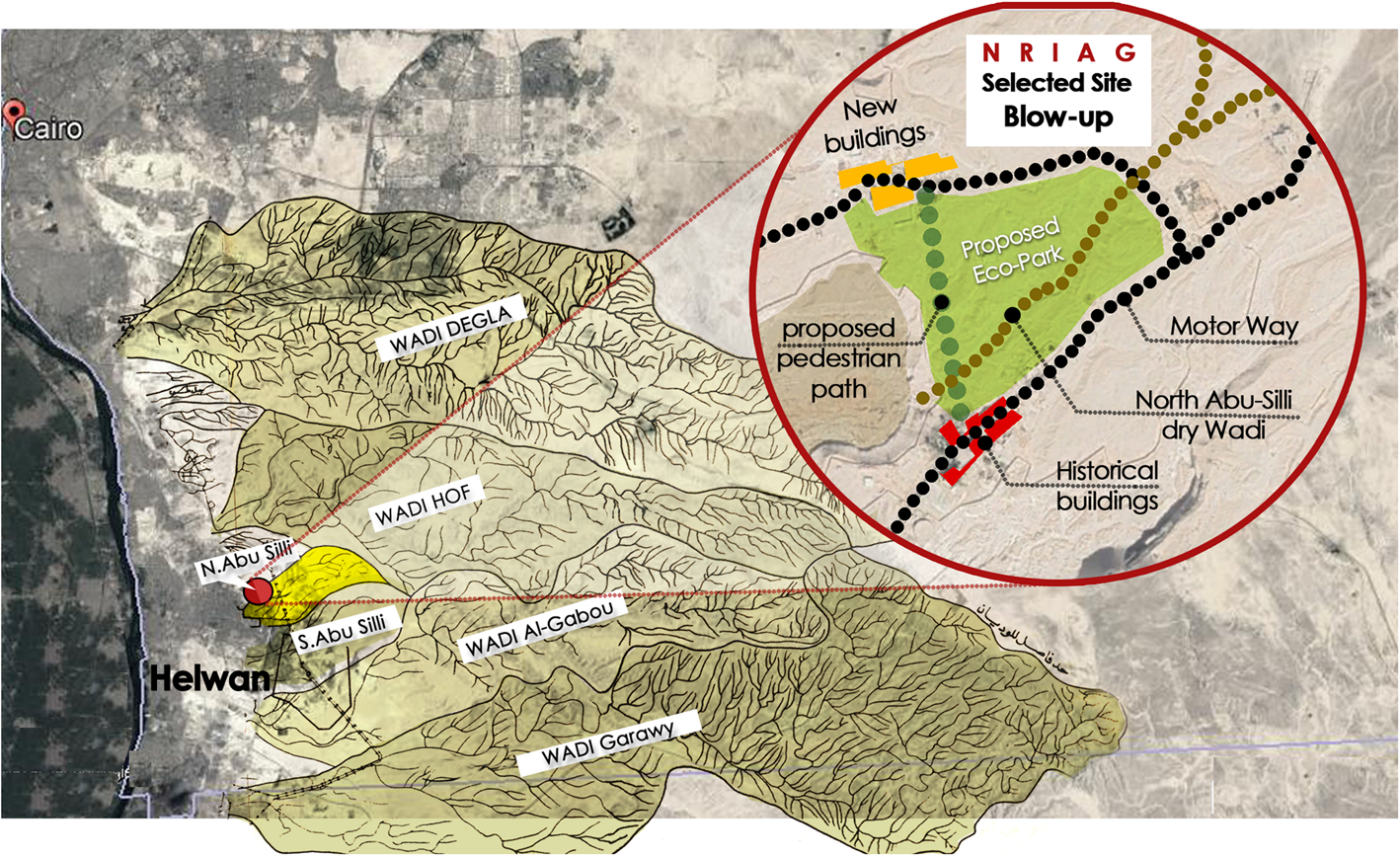
Google satellite image showing the 6 Wadis of South-East Cairo, focusing on NRIAG site. Source: Authors, adapted based on [18]

Many Wadis are concentrated in South and East Cairo, mainly in the Helwan district. Helwan was established in 1868 under the rule of Khedive Ismail as a therapeutic winter destination for Egypt’s notables, depending on its famous sulfur springs. This therapeutic function started to decline with the establishment of the Tora and Helwan cement factories. During the Nasser era (1952 to the late 1960s), the government decided to transform Helwan into a centre for heavy and military-strategic industries. Since then, Helwan has become one of the most polluted areas in Cairo [16]. Exposure to airborne particulate matter generated from the cement industry continuously threatens people’s health in Helwan, causing respiratory inflammation, chronic allergies, lung cancer and sleep disorders [17]. These problems elevate the necessity of restoring nature in Helwan. Thus, the researcher decided to focus efforts on improving a segment of Abu-Silly Wadi in Helwan [18]. This Wadi lies on the National Research Institute of Astronomy and Geophysics (NRIAG) property, as emphasised in (Fig. 1), Helwan Wadis.

### NRIAG site in Helwan

The NRIAG site in Helwan was selected for the eco-park project, as it meets the selection criteria. Some of the historical buildings of the institute are on the tentative list of the world heritage site in science and technology, which add value to the selected site [19]. The historical buildings are separated from the new campus by a dry Wadi, and they are only connected with a long motorway to avoid the topographical change of the Wadi. Thus, the need of NRIAG’s employees for a shaded pedestrian shortcut between the two campuses provides seed to the eco-park proposal, as seen in the blowup of the NRIAG selected site (segment of Abu-Silly Wadi) (Fig. 1). Furthermore, The institute offers a broad spectrum of scientific researchers and high valued experts in Astronomy, Geophysics and Meteorology. NRIAG is frequently involved in scientific community services, related to school and university students in addition to astronomy and science enthusiasts.

### Scope of this research

This exploratory study is an initiative call from a volunteer landscape architect and a junior urbanist interested in Wadis who have a vision for an eco-park at the NRIAG site. The collaboration of multidisciplinary experts, the local community and non-governmental organisations (NGOs) are recommended to join the design team, after the first brainstorming session. Due to the high cost of the study, a small grant is proposed to support the focus group workshop and some experts’ technical reports. The study is part of an ongoing project, and this research is the first phase that aims to propose activities and zoning design.

Broad strategic goals of environmental, educational, economic and social development are expected even before the workshop takes place, and the value of each goal is investigated later. The strategy of education and raising the scientific and environmental knowledge, for example, is not only achieved by the establishment of the park but is planned to be a continuous activity during the participatory design process. This is to be achieved through exchanging knowledge, having discussions, and even occur through the construction phase. During this process, mock-up workshops with voluntary experts and local labours shall build experience and provide job opportunities.

## Methods

Researchers think that many methods and tools already exist in the ecodesign field, yet other tools remain a search topic and still expand [15]. We argue that although eco-park design is the landscape architect’s crucial role, the engagement of other perspectives and multi-disciplines in the early design ideation process is also important but very challenging. Nassauer [4] declares that designing a single shared landscape usually engages groups with different backgrounds and languages, such as designers, scientists, technical experts, local stakeholders and policymakers. In such a practice, landscape functions are conceptualised as a boundary object that allows different groups to cooperate in “back and forth” iterative negotiations. This ideation process needs systematic and continuously evolving tools and strategies to be adopted according to the changeable situation.

Multi-methods of data collection, analysis and formulating solutions can be used to enrich the eco-landscape design. This is important especially in developing countries, complex situations and the arising post-pandemic crisis. Such diverse forces and different backgrounds need comprehensive, collaborative multi-methods when dealing with the hybrid eco-landscape design process. This practice is hardly applied in Cairo, where the landscape work is usually the landscape architect’s responsibility. Even though participatory involvement is rarely found in the early stages, as in the notable projects of the cultural park for children in Sayyida Zeinab and Al-Azhar Park.

Therefore, this paper’s main objective is to investigate how different participatory approaches, like individual meetings, small group work, focus groups workshops and distributed work packages tactics, can help in eco-landscape design, decision-making and management, when combined in one conceptual framework with other digital tools, as modelling and GIS analysis.

### Preliminary individual meetings

The first brainstorming session recommends the collaboration of multidisciplinary experts. Employees in NRIAG and specialists in land surveying, microclimate, soil and geophysics, besides other experts in planting, irrigation and energy, are asked to join the research. NGO and the local community are recommended too. The researchers conduct site inventory and preliminary analysis, then qualitative tactics of twelve preliminary individual meetings and semi-structured interviews with the experts and the stakeholder are followed to investigate the possibility of cooperation, collect primary data and raise the issue of multi-method techniques to improve coordination.

According to each engaged discipline, the primary types of collected data vary between qualitative and quantitative. Quantitative data are mainly derived from scientific factors and site conditions, while qualitative data are derived from historical background, local community needs and proposed activities. Some of the meetings’ output are technical reports of microclimate data, earth’s surface and subsurface properties, surveying map of contour lines, soil report, environmental data, archival documents, species characteristics and plant types. This diversity of data highlights the need for new analysis strategies and addresses the need for multi-methods analysis.

The preliminary meetings highlight essential issues. One of the raised threats is the poor water infrastructure on the site, which hardly satisfies the employees’ needs. This issue escalates the importance of treating wastewater and calls for specialists in this field. An NGO with practical experience in building youth capacity in wastewater treatment is selected. Constructed wetland and septic tanks are recommended as natural processes for grey and black water ecosystem treatment. Recycling construction waste and using earth material are proposed to the hardscape. The most valuable potential outcome of these efforts is the availability of valuable equipment and scientific tools in NRIAG, besides the experts who can share their knowledge. These potentials highlight the institute’s role in educating and raising scientific awareness that worth being widely and openly exhibited in the park.

### Coordination and group meetings

Following the individual meetings, six small coordination group meetings are organised by the researchers to discuss conflict issues, for example, the soil type that affects planting species selection, infrastructure and the alternatives of wastewater treatment, the chosen list of plants that respond to the water amount and wastewater irrigation system and employees’ need versus site problems and potentials.

Many raised issues and conflicts are solved. For example, greywater treatment is valued, and the drip irrigation system is proposed. Accordingly, drought-tolerant plants are listed to moderate urban poverty’s environment. These group meetings faced some misunderstandings and misperceptions between the diverse backgrounds. Comprehensive coordination among the involved participants is therefore suggested.

### Focus group tactic as a participatory approach

The small coordination meetings are followed by two focus group workshops to present the project progress, facilitate cross-disciplinary data transmission, conduct broad coordination, guide the participants to work on the project objectives, propose activities and widely engage in discussing zoning plans.

Although the focus group is a qualitative tactic of data collection method that is generally used in the social sciences [20], there is a further reason for using the focus group method. This is its inhere participatory nature based on group processes [21, 22]. Nevertheless, it is expected that some multi-method proximity and discussions may trigger anxiety. Thus, as Sendall [23] stated, “It is important researchers are cognisant of and learn from potential tensions within research teams due to juxtaposed philosophies, methodologies and experiences”. Hence, the importance of participatory action through a focus group workshop is to minimise the tension and reduce misunderstandings and biases between the different qualitative and quantitative teams to reach maximum coordination [23]. As Kreuger [24] argues, focus groups are “not to infer but to understand, not to generalize but to determine the range”.

It is worth mentioning that participants are asked to engage in focus groups for the following reasons: their expected contribution, their concerns with other engaged members and their interest in the research field. Participants are not selected according to their gender bias or ratios. The researcher usually “facilitates” the focus group discussions to deepen the understanding and “moderates” the interdisciplinary coordination [20]. On this account, focus groups influence how complex situations are perceived and dealt with.

## Results

### Exploring the outputs of the focus group

The first workshop investigates the participants’ degree of involvement with the site, how they use or expect to use the surrounding environment, their suggestions for new activities and their vision for the future. Thus, the aim is to propose the possible activities that can take place in the selected site. These activities are proposed in the first brainstorming sessions and individual meetings, but elaborated according to several factors. These include the needs of the local community, NIRAG employees and what each participant or expert can offer based on his/her experience and dreams. Participants set a list of activities, which are discussed, evaluated and sorted according to priority. Alternatives of these activities are allocated on a site physical model during the focus group workshop.

**Fig. 2:**
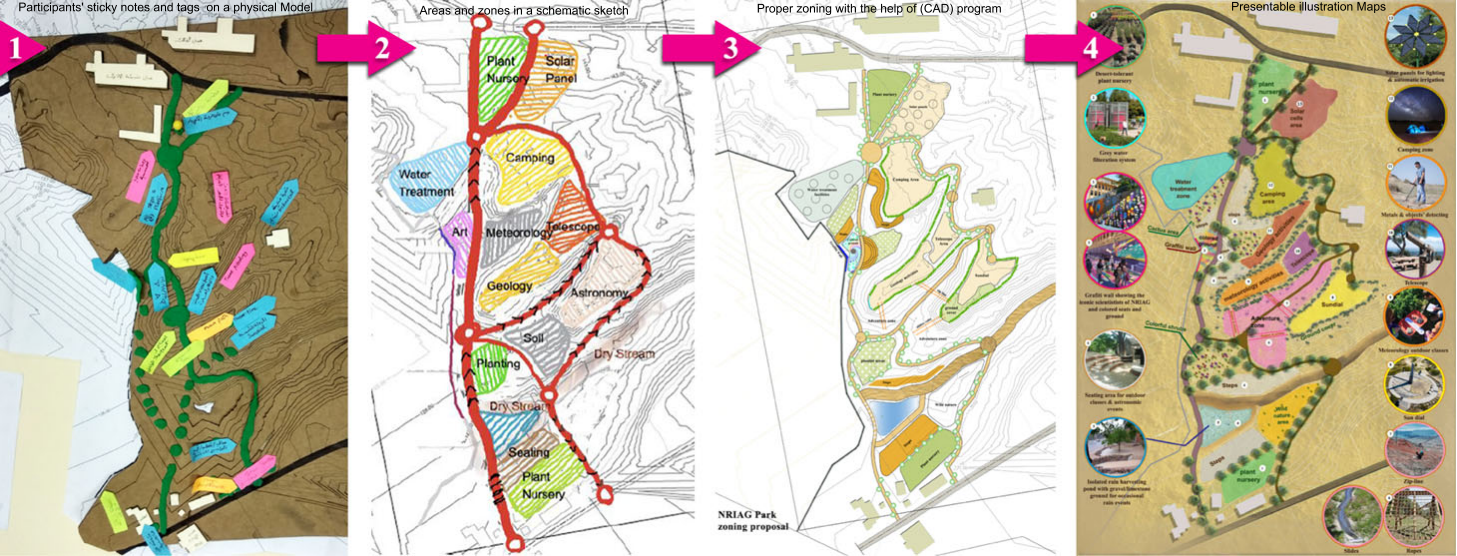
The modelling progress of the proposed activities on the eco-park site

The activities are assigned through a progressive evolution, as demonstrated in (Fig. 2), using sticky notes, tags and pins on a physical model of the site’s topography and surrounding context. These sticky notes are translated into areas and zones in a schematic sketch, then transformed to proper zoning alternatives, with the help of the survey levelling maps, using the computer-aided design (CAD) programme. Later, after having had many discussions and multiple modifications, the researchers clarify the maps on presentable illustrations, including similar precedent visualisation and images to support the perceivers’ perception and the ideation process [25].

Participants contribute to the workshop outcomes by engaging in discussions to achieve the best data synergistic interaction. For example, a path’s form and shape are set about the users’ needs and traced from the employees’ movement analysis. The main path is a shortcut connecting the old and new buildings. The secondary path is more prolonged, mimicking the original contour lines to maximise exposure to the activities, attract children and teenagers and provide a wheelchair-friendly gradient ramp. The same can be said about other necessary and optional activities, such as exploring educational instruments and sitting areas.

### GIS role in adapting the preliminary zoning plan

One of the results of the first workshop is the joint GIS specialist. The GIS programme is commonly integrated with urban and planning research from the early stages. It is usually used as an analytical tool that helps design decisions, where added algorithms of different variables can supply the demand points efficiently.

This integration of modelling and GIS databases [26] contributes to design development, scientific decision-making and the best allocation for proposed activities [27]. GIS provides more potential for coordination when all maps are saved in shapefiles and KML formats as more data can be available for all experts. These maps help in adjusting the design as per added factors and conditions.

Here, GIS is combined with other participatory approaches to merge the mixed resultant qualitative and quantitative databases to facilitate the design progress and mutual learning [4]. For example, the precipitation data, specifically in exceptionally severe weather, such as the heavy rain that took place on the 13 March 2020, are estimated and combined with other data, such as topographic contour lines, geophysics data, the layer of water flow directions and other analyses of the dry stream. The focus group discusses the possibility of catching the rain and proposes the eco-construction method, while the GIS analysis coordinates the previous merged layers and provides guidelines to locate the rain harvesting pond at the best point. Although it rarely rains in Egypt, the project looks to maximise the use of rain and search for several sources of irrigation besides the wastewater treatment, where every single drop of water counts. We argue that GIS tools play a significant role in comparing alternatives, helping designers in scientific-based decision-making and adapting the finalised design.

Another example, constructing a telescope pier is one of the proposed observatory activities recommended by the NRIAGs astronomer. But checking the locations at the highest altitudes on the site and the best location of the telescope, checking the surrounding landmarks and adjusting the soft-scape is not easy to retrieve without the help of GIS. The telescope is best allocated according to the surrounding site conditions and the shed-view angle of the landmarks in the broad context.

**Fig. 3:**
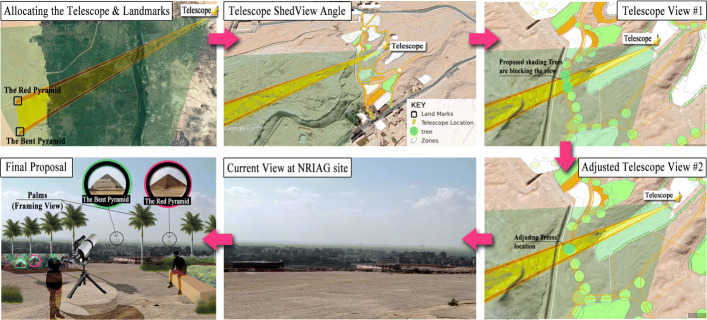
GIS tool contribution in developing the landscape design

By locating the bent pyramid and the red pyramid at Dahshur on Google Earth and with GIS Data Elevation Modelling, some proposed shading trees on the main path are removed to unblock the view. Later, the landscape architect recommended framing the view with palms instead of trees, to widen the view. We argue here that integrating the GIS tool with the participatory approach contributes to the accuracy of landscape design, as Fig. 3 elaborates.

### Evaluating the outputs

**Fig. 4:**
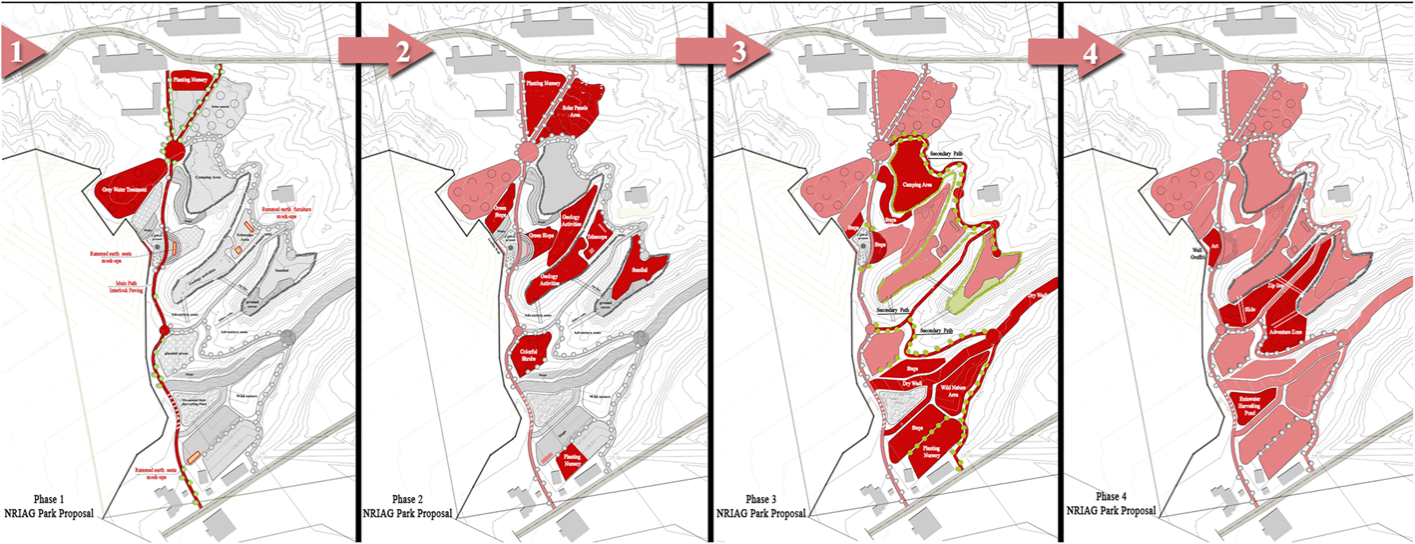
Recommended phases of execution

Due to the COVID-19 lockdowns, the second focus group workshop is conducted online, on which the zoning map is presented to the participants. The eco-landscape design process is continued; a range of quantitative analytical strategies is integrated and online forms and surveys of the projects’ strategic goals are done. The participants’ weighted voting of each goal is analysed, the number of participants’ votes which agree on the proposed activities is counted and votes on the priority of execution and best practice are evaluated. Accordingly, the implementation cost analyses are computed. Later, the analysis of these numerical data is translated into different phases of zoning plans, where the shortcut path, the planting nursery and the wastewater treatment station are prioritised. Other activities are sequentially suggested to occur, as shown in (Fig. 4).

### Integrating multi-methods in a conceptual framework

**Fig. 5:**
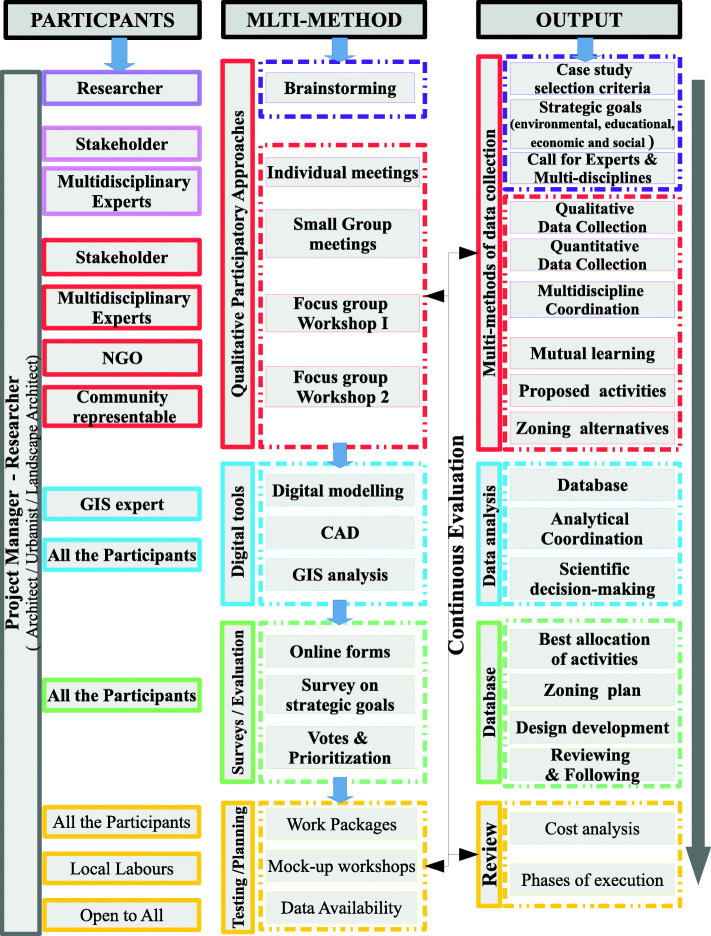
The eco-landscape integrative multi-methods framework

Researchers assert that combining multiple scopes and perspectives, implementing the mixed method of collecting a wide range of qualitative and quantitative data and the complexity of using multi-methods and various tools of analysis within participatory action research match ecodesign research and practice. They advocate that integrating modelling and databases supports eco-design. Proposing applications as CAD brace design activities and the importance of integrating both modelling and CAD programmes with GIS analysis outcomes is revealed [26] to help design scientific-based decision-making. This complex environment of multi-methods yields the construction for a conceptual framework to join all participants’ presentations of the varied collected data, shared ideas, discussions of needs and possible outcome activities in addition to the evaluations, all in a broad sense. Thus, the answers are demonstrated sufficiently instead of obstacles [28], as explored in the following conceptual framework (Fig. 5).

### Planning the work packages and the following phases

Although this paper presents the first phase of an eco-park project, as previously elaborated in the individual meetings, group coordination, focus group workshops, modelling, digital programmes and GIS integration, the researchers set the plan for the following phases: mock-ups implementation, detailed design, working drawings and constructing the design elements based on their priority. Moreover, the research is based on distributing the tasks among experts and work packages [29] as this is a crucial concept in project scope management.

This project is broken into ten main work packages, such as wastewater analysis and treatment, planting, recycled and rammed earth construction, energy and environmental monitoring. Each work package has targets and outputs. All work packages are managed and coordinated by the project management board. The researchers, whose backgrounds in landscape architecture enable them to lead the dissimilar teamwork, establish ground rules for the group, map the different activities scenarios. Finally, with the help of GIS, produce an optimum ecodesign solution for the selected case within the complicated situation and limitation.

It is agreed in the workshops that each work package is responsible for a set of tasks. For example, landscape architects, based on the participatory workshops, address designing themes and functional activities, find creative and interactive ways to expose the wastewater treatment facility and prepare a detailed landscape of each zone in the park. Planting and soft landscape experts list the most suitable plants to the soil type, limited water source or the treated wastewater drip irrigation system. Planting experts also create nurseries to provide the site with needed green areas and conduct participatory planting workshops with the local community. Energy experts provide and install solar panels to feed night lighting, electrical outlets and automated irrigation systems. Wastewater treatment expert produces greywater treatment and reuse systems (GTRS) and conducts participatory workshops to build local workers’ capacities to carry on the future maintenance. Experts in recycled and rammed earth construction conduct a series of participatory workshops using construction waste and upcycled waste materials with local workers to construct the following, hardscape, signage, seats, furniture, playgrounds and educational instruments.

**Fig. 6:**
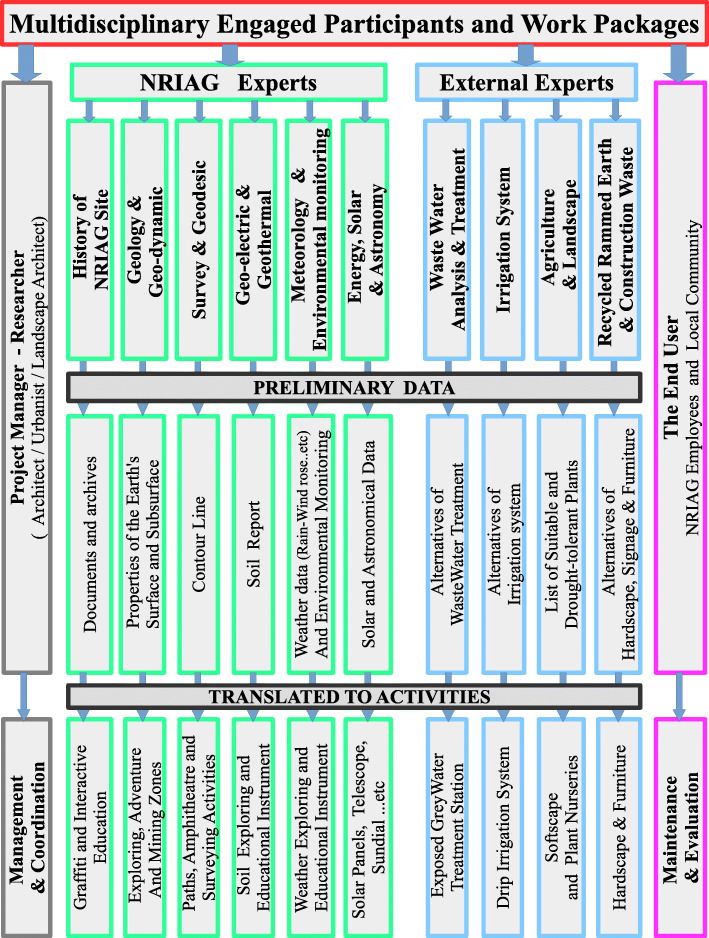
Multidisciplinary engaged participants and work packages

The distribution of the tasks among work packages, as shown in Fig. 6, helps in the execution phase and the project management, where each work package hires the most talented experts and local labours. It also controls and manages separate flexible working hours to reduce labours on site.

**Fig. 7:**
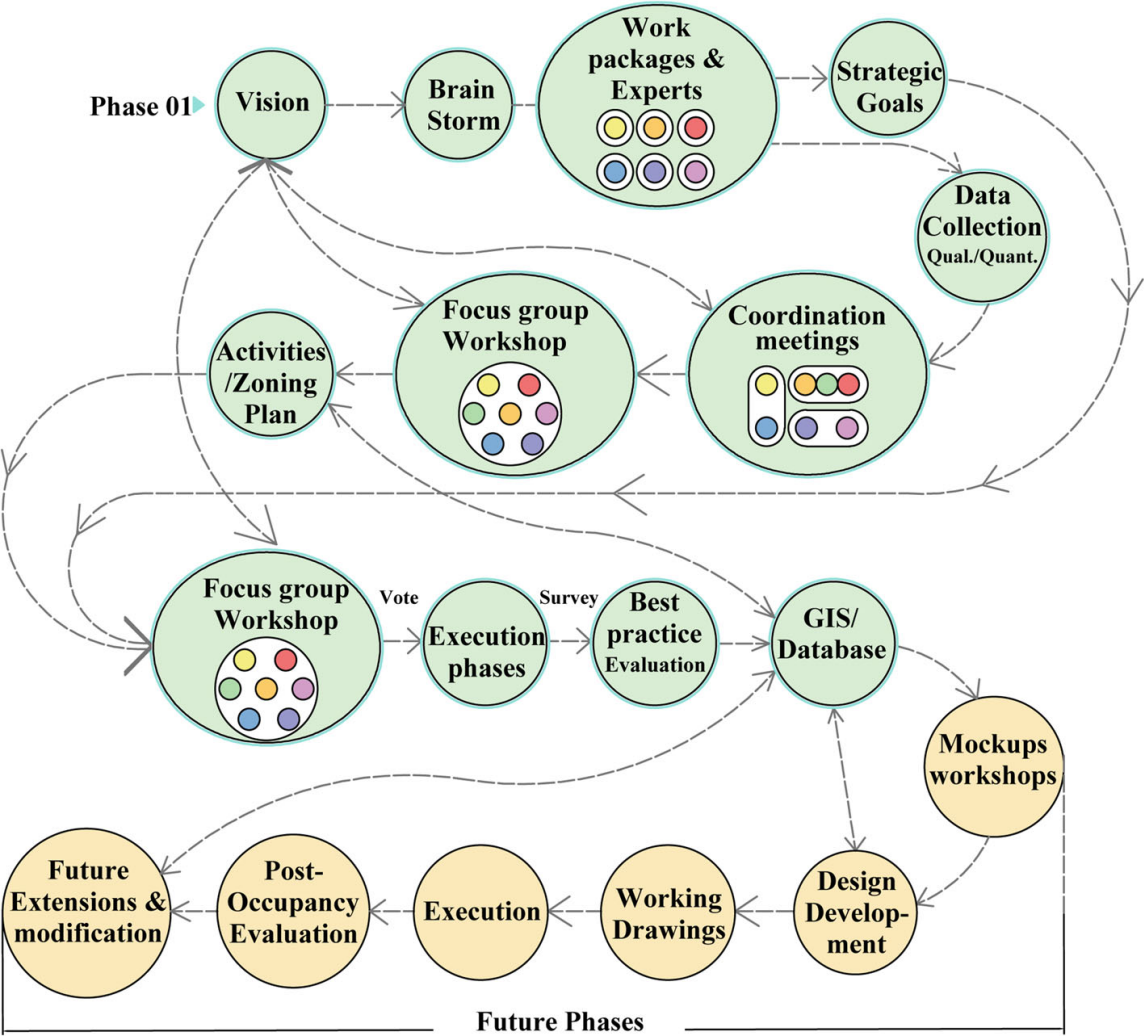
The NRIAG Park participatory design process

The phases mentioned above shall be followed up by continuous monitoring and control. Post-occupancy evaluation is recommended to allow design modifications and desirable future extensions. These future phases of the research are summarised in the design process plan, as shown in (Fig. 7).

## Discussion

This project provides a model for the eco-landscape design process, methods, database and a conceptual framework. This shall face the complex environmental, economic and social challenges and guide the landscape architect in the subsequent detailed phases. We argue that an integrative multi-method framework is a fundamental approach to transforming current environment-stressing patches or spaces into multi-functional natural ones that blend with local ecosystems and contribute to community development.

We appraise the participatory approach in the eco-landscape design process. It enables the landscape architect to integrate the multi-disciplines in the principles of sustainable development. Although the focus group tactic is a social science concern, it adds dynamic aspects to the ecodesign process and the idea generation. In this case, the focus group workshops add value to the participatory research notion, engage the multidisciplinary participants in the design process and encourage them to develop strategies that sustain the project. When the researcher is part of the focus group and not an outsider, they play a double role: a facilitator and observer. The researchers’ dual role helps them run the discussion smoothly, consider the load weight of the participants’ votes according to their involvement, make sure they deeply understand the aim, control time and reorient the discussion when it drifts out of the research target.

Our observation, however, is that the nature of the data produced, when mapped on a physical model, contributes to the ideation process. We argue that modelling cultivates qualitative data analysis in a time-saving and creative process when compared with the traditional analytical way. The traditional way usually depends on taking notes and suggestions of the participants, transcribing and coding them, then the landscape architect leads the way in translating the collected codes and activities into a zoning map form. Integrating digital modelling and GIS in earlier phases, however, helps in decision making and placing activities in the proper location. We argue here that adding GIS can potentially adapt the preliminary design and keep both the scientific base and the participatory spirit. As a consequence, GIS involvement in detailed future scenarios is recommended.

We argue that if the resultant framework and the workshop products are available sources, they will widely share data, limit the barriers, encourage the public to develop the process continuously and facilitate voluntary action and fundraising opportunities.

Since this eco-park is based on a participatory citizen design approach, no one is excluded. The initiative workshop is a call from young women landscape architects, as women and older employees in NIRAG compose nearly half of the workshop participants. The science park already targets students in nearby schools and Helwan University. The main shortcut path is designed to encourage walkability, and the paths’ slopes are designed to suit disabled and older employees. The potentials above meet the Sustainable Development Goals (SDGs), Egypt 2030 development strategy [30] and the community’s need for such public spaces. Hence, such opportunities support the sustainability of the project.

The lack of information and the absence of transparency in data sources of the fauna, flora and wildlife ecological database are some of the challenges facing such research, and it is a questionable concern in Egypt [31, 32]. Lack of practical experience is shown when both the expert and the stakeholder reject the exposed wetland proposal, as wastewater treatment due to worries of sewage odour and maintenance concerns. However, the wetland is a natural solution widely used and treated in other countries with low maintenance costs.

Although technology risk is minimal as this project adopts a simple eco-friendly approach, time risk is crucial, especially in a lockdown. We argue that time can be reduced by distributing the tasks among work packages that can work in parallel at different zones. The distributed management guarantees an increase in productivity, manages separate flexible working hours, hires the talented local worker and closer to sites and reduces labours on-site, all that with fewer overheads are very beneficial in post-pandemic management. Maintenance risk is diminished by conducting a series of participatory workshops to build the capacities of the local workers. Moreover, the engagement of the local community provides job opportunities and direct evaluation instruments.

The Covid-19 lockdown causes a delay in the work progress; it allows a limited number of physical meetings with fewer participants when urgently needed, while the rest of the meetings and workshops are conducted online. Nevertheless, we argue that virtual workshops control time with few interruptions. Also, voting using online forms permits researchers to collect information, store feedback, facilitate analysis productivity and disseminate information on the planning progress and problems encountered. This complex work environment turns the critical eye on not only the project’s importance and effect on physical health and mental wellness, but also the contribution of this broad multi-methods framework to the eco-landscape design process. This process offers integrative potentials, especially when dealing with the current complex situation in developing countries.

## Conclusions

This study addresses a unique case. Firstly, the site location in a dry Wadi gives it a great potential to integrate such a marginalised area with the compact urban fabric. Secondly, its position between NRIAG’s historic buildings and the new campus offers a strong presence of experts who can play a significant role in community development, educate science and raise environmental awareness. Thirdly, the proposed eco-park framework can catalyse development at different pockets and sites in a city-wide short and long-term action plan through continuous research and ongoing prototypes on the ground. This prototype may distribute information about implementing the know-how of one or more of the work packages to the nearby sites. It may supply and exchange the used moulds and equipment, provide trained labourers and experts’ technical advice, host the indigenous plants in its nursery and propose different themes and functions according to each site situation, potentials and needs.

The shortage of information and the lack of practical experience are some of the challenging issues facing eco-design in developing countries. Funding is also one of the main problems facing eco-design projects, especially after the COVID-19 outbreak, which triggered a severe economic crisis that forced the government to cut the budgets of many services. Therefore, we argue that the research needs to involve more specialists such as ecologists to provide a more comprehensive understanding of local ecosystems’ functions, flora and fauna and economists to assist in fundraising. We also recommend that psychologists emphasise and analyse the used language and promote communication skills between the diverse teams.

We suggest more deep studies on linking issues like water scarcity, crowding and searching for new opportunities and new local eco-conscious methods that need to be integrated to understand the complexity of all the factors at play within the current process. In-depth investigations need to be carried out using comprehensive frameworks such as the one proposed to restore the natural environment and human wellness without stressing limited resources.

We finally assert that comprehensive partnerships between local government and municipality as well as the educational and research institutions, presented by Helwan University and NRIAG, NGOs and the civil society, with the private sector, are essential to achieve coherent Eco-design.

## Data Availability

The data that support the findings of this study are available from the multidisciplinary experts, NRIAG’s researcher and BENAA foundation. However, restrictions apply to the availability of these data, which were used under license for the current study, and so are not publicly available. Data are, however, available from the authors upon reasonable request and with permission of [the multidisciplinary experts].

## Abbreviations

CAD: Computer-aided design
GIS: Geographic Information Systems
GTRS: Greywater treatment and reuse systems
NGO: Non-governmental organisation
NRIAG: The National Research Institute of Astronomy and Geophysics in Helwan
SDGs: Sustainable Development Goals
